# Evaluation of coupling and coordination, obstacle diagnosis, and optimization pathways of medical–preventive integration in primary healthcare institutions in Hebei Province from 2017 to 2024

**DOI:** 10.1371/journal.pone.0351931

**Published:** 2026-06-30

**Authors:** Botao Yang, Kexin Li, Zike Zhao, Zhihui Cao

**Affiliations:** 1 School of Economics and Management, North China University of Science and Technology, Tangshan City, Hebei Province, China; 2 Center for Health Policy and Management Research, North China University of Science and Technology, Tangshan City, Hebei Province, China; Central University of Finance and Economics, CHINA

## Abstract

Medical–prevention integration constitutes a core governance strategy for advancing the Healthy China initiative, with primary healthcare institutions serving as the foundational delivery platforms for its implementation. To systematically evaluate the development level, spatiotemporal evolution, and key constraining factors of medical–prevention integration at the primary level, this study takes all township health centers and community health service centers in Hebei Province as the research sample. Drawing on panel data from the Hebei Health Statistical Yearbook covering 2017–2024, an evaluation index system for the coupling and coordinated development of primary medical services and public health services was constructed and authoritatively validated using a modified Delphi method. Indicator weights were determined through the entropy method, and an integrated quantitative analytical framework was employed, incorporating the coupling coordination degree model, the relative development model, and the obstacle degree model. The results indicate that primary medical–prevention integration in Hebei Province exhibits a typical pattern of “high coupling but low coordination”. Although medical and public health services have established a relatively stable institutional linkage, their overall coordinated development remains at a persistently low level, with medical services generally outperforming public health services. Township health centers demonstrate significantly higher composite development levels and coupling coordination degrees than community health service centers, forming a pronounced urban–rural dual structure in primary-level medical–prevention integration. Furthermore, substantial heterogeneity exists between the two types of institutions in terms of relative development structures and core obstacle factors. Lagging public health service capacity, along with misaligned financing and incentive mechanisms, constitutes the principal bottleneck constraining high-quality development of medical–prevention integration. By uncovering the underlying structural and institutional tensions within primary-level medical–prevention integration, this study provides robust empirical evidence and policy-relevant insights for optimizing integration strategies and advancing the construction of an integrated healthcare delivery system in Hebei Province and other comparable regions in China.

## 1. Introduction

Medical–prevention integration represents a central health governance strategy in China’s advancement of the Healthy China initiative. Rather than an isolated policy construct, it has evolved from earlier paradigms such as the integration of prevention and treatment and the coordination between medical care and public health, with its connotation continuously enriched through policy development and practical implementation [1]. At its core, medical–prevention integration seeks to dismantle institutional and functional barriers between curative medical services and public health services, thereby achieving an organic integration of clinical treatment, disease prevention, and health promotion. This strategy is designed to address multiple contemporary challenges, including the rising prevalence of chronic diseases, persistent infectious disease threats, rapid population ageing, and widening health inequalities. By fostering an efficient, equitable, and sustainable integrated healthcare delivery system, it ultimately aims to improve population health outcomes [2]. The concept is therefore of critical significance for optimizing health resource allocation, enhancing service efficiency and equity, and strengthening the public health system.

Primary healthcare institutions constitute the cornerstone of China’s healthcare delivery system and serve as the essential network ensuring equitable access to basic medical and public health services for both urban and rural residents. These institutions mainly include township health centers and community health service centers. In addition to providing diagnosis and treatment for common and frequently occurring diseases, they undertake essential public health services, health management, and disease prevention and control responsibilities [3]. As the “first line of defense” safeguarding the health of hundreds of millions of residents, the development and institutional robustness of primary healthcare institutions are fundamental to the realization of the Healthy China strategy.

In September 2025, the National Health Commission of the People’s Republic of China led the formulation of the Implementation Plan for the Primary Healthcare Strengthening Project, which explicitly identified the enhancement of medical–prevention integration at the primary level as a core task. The plan emphasizes strengthening the synergistic integration of preventive and curative services within primary healthcare institutions, optimizing service delivery models and resource allocation efficiency, and further consolidating the grassroots health protection network. Through advancing the implementation of the hierarchical medical system, the initiative aims to provide urban and rural residents with more equitable, accessible, continuous, and high-quality basic healthcare services, thereby reinforcing the primary-level foundation of the Healthy China initiative.

## 2. Literature review

### 2.1. Types and practical explorations of primary-level medical–prevention integration models

Domestic scholars have conducted extensive theoretical and empirical explorations of medical–prevention integration models. At the theoretical level, substantial research has focused on clarifying conceptual definitions, elucidating underlying mechanisms, and constructing analytical frameworks and indicator systems for evaluating integration performance [4]. In practice, China has developed several regionally representative models of medical–prevention integration. These include the Luohu Hospital Group model in Shenzhen, the county-level medical consortium models in Jieshou of Anhui Province and Rongchang of Chongqing, the “trinity” management model in Yunnan Province and Xiangshan County of Ningbo, and the “Three-in-One” model in Yangcheng County of Shanxi Province [5]. Drawing upon successful experiences such as the family doctor system, scholars have further proposed context-adapted integration models suited to China’s institutional environment [6,7].

Furthermore, research focusing on chronic disease management and functional integration has yielded substantial progress: Dou Rongrong demonstrated through comparative analysis that county-level Chinese medicine hospital medical communities possess significant advantages in standardized management rates [8]. Ma Liping proposed a primary chronic disease integration model driven by optimized incentive mechanisms, information interconnectivity, and the tilting of medical insurance payments toward primary care [9].Li Minggang, within the context of “synergetic management of three highs and joint prevention of six diseases,” established a “seven-integrated” health management service path centered on family doctor teams [10]. Additionally, in the direction of organizational structure and referral mechanisms, Zhou Xufeng summarized the “integrated” management experience of chronic diseases through public health talent secondment and school-local collaboration [11]. Chen Hongying explored a new primary integration model by strengthening three-level hierarchical management, which effectively improved the control rates of chronic diseases [12]. Ding Xia, based on integrated health service theory, constructed a specialized medical-preventive integration model for thyroid diseases [13]. Sun Jian innovatively developed a “One Body, Two Wings” service model, enhancing management efficiency through patient stratification and the coordination of preventive and medical functions [14].

In the international context, integrated medical services predominantly utilize common primary chronic diseases as entry points, focusing on the synergy between legislative guarantees, technical interventions, and resource allocation [15]. In the direction of policy legislation and administrative integration, the U.S. Affordable Care Act eliminated interstate and regulatory barriers through preventive service mandates, ensuring access to evidence-based preventive services for the vast majority of the population [16]. Northern Ireland established an administrative foundation for the systemic integration of preventive and public health services by legislatively transferring school health services to the health department [17]. In terms of clinical intervention and evidence-based practice, a cluster randomized controlled trial by Dubey proved that the use of evidence-based, gender-specific preventive healthcare checklists in family medicine clinics significantly increased the delivery rate of preventive services during adult health examinations [18].Regarding technology-driven innovation and resource optimization, research has achieved breakthrough progress: Kothinti demonstrated the potential of artificial intelligence and Long Short-Term Memory (LSTM) networks in predicting chronic disease progression to support personalized interventions [19]. Khakimova and Barotova emphasized the central role of preventive medicine, advocating for the integration of prevention and treatment through health education and prevention programs to reduce morbidity and mortality rates [20]. Finally, studies on regional disparities and full-cycle management indicate that while the public health functions of medical institutions vary across regions, the use of big data, clear division of labor, and technical collaboration can effectively mitigate resource shortages and achieve integrated management throughout the entire life cycle of chronic diseases [21–23].

### 2.2. Current status and major challenges of primary-level medical–prevention integration

From the perspective of domestic research, the academic community has focused on analyzing the contradiction between rapidly evolving medical demands and rigid institutional structures, identifying challenges across macro-systemic, micro-human resource, and operational dimensions. Regarding healthcare systems and functional positioning, research indicates that amidst population aging and the epidemiological transition, institutional barriers between medical and public health systems, the marginalization of public health functions, and resource imbalances have become primary obstacles to integration [24,25]. In terms of human resources and professional competency, progress has been made in investigating staff adaptability: Li Lifen revealed deep-seated issues such as the acute shortage of general practitioners and uneven personnel distribution through an analysis of qualifications and practice status [26]. Zheng Zhe further identified a “mutual knowledge gap” caused by independent working mechanisms, wherein clinical staff lack public health expertise and public health personnel lack diagnostic capabilities [27].

In the international research arena, scholars have explored the systemic drivers of integrated services and the barriers to implementation across diverse resource contexts. Concerning systemic integration strategies and theoretical mechanisms, research underscores the necessity of transitioning from fragmentation to synergy to ensure continuous health protection [28]. Rechel explored the systemic, organizational, and interactive factors influencing integration, proposing five major strategies and corresponding policy options to provide a theoretical basis for institutional optimization [29].

Regarding practical challenges in resource-constrained settings, research reveals a more severe reality: Wendimagegn and Bezuidenhout’s study in Ethiopia demonstrated that extreme shortages in skilled manpower, equipment, and medication—coupled with a clinical bias toward treatment over prevention and low patient health literacy—have resulted in exceptionally low integration rates in primary care [30].

### 2.3. Influencing mechanisms and optimization pathways of primary-level medical–prevention integration

In response to the challenges encountered by primary healthcare institutions in advancing medical–prevention integration, scholars have proposed a range of institutional and operational reform strategies. These include promoting institutional integration between the medical service delivery system and the public health system, exploring the introduction of alternative health insurance payment mechanisms, and improving remuneration and incentive structures for public health personnel [31]. Broadly, the literature concentrates on: organizational and institutional integration—including institutional alignment, graded management and referral mechanisms, and the use of insurance payment and incentive levers to shape provider behaviour; human resources and capacity building—emphasizing the development of multidisciplinary family-doctor teams and increased, targeted training for primary care staff; resource allocation and performance constraints—focusing on staffing, compensation and incentive structures that affect motivation and service supply; and information and governance elements—promoting interoperability and resolving fragmented, multi-headed administration to secure the implementation of integrated services. Empirical and policy work has progressively moved from problem description to quantifying mechanisms and testing key factors. For example, proposals for integrated service systems and talent-development pathways have been advanced [32]. Fan Boyang in a study of primary care physicians in Shandong Province, found that female physicians, those with junior college education, higher levels of cognition regarding medical–prevention integration, and supportive institutional environments exhibited stronger behavioral intentions to deliver integrated chronic disease services, highlighting the combined effects of individual characteristics, cognitive factors, and organizational context [33]. Zhang Huifang reported that physicians with undergraduate or higher degrees, familiarity with integration policies, strong institutional support, and repeated training demonstrated superior service capacity; they recommended enhancing policy dissemination, targeted training, and innovative performance appraisal mechanisms to improve professional competence [34]. Yu Tian, based on surveys of township health centers in two provinces and four cities, identified adequacy of staffing levels, social recognition, and perceived fairness in salary and welfare distribution as key determinants of work motivation among primary healthcare workers [35]. Fan Wenyu, in research on family doctor teams in Shandong Province, showed that educational level, professional title, knowledge of integration policies, institutional emphasis, and training frequency significantly influenced team collaboration performance [36].

At the governance level, Gu Hai argued that overcoming fragmented administration and structural separation requires mechanism innovation across organizational management, service delivery, health financing, workforce development, and information interoperability [37]. Cui Zhaohan, examining tightly integrated county-level medical consortia, found that environmental and individual factors jointly shape healthcare workers’ willingness to engage in integration practices, with team-level integration exerting the strongest influence; moreover, the influencing mechanisms differ across county-, township-, and village-level personnel [38]. Lai Qingling, drawing on the Horn–Mitchell policy implementation model, analyzed integration policy implementation across six dimensions—policy goals and standards, resources, implementation approaches, characteristics of implementing agencies, system environment, and implementer value orientation—identifying vague policy objectives and resource insufficiency as primary barriers [1]. Hu Meili, through qualitative comparative analysis of 15 cases, concluded that multi-level and meso–micro integration are effective mechanisms for improving integration outcomes, with service integration serving as the foundational guarantee; however, policy indicators for system integration and professional integration require further refinement [39].

International research complements domestic work by strengthening the nexus among evidence, instruments and context, and by supplying methodological and policy lessons for China. Empirical studies indicate there is no single best model for integrating prevention and care; institutions typically adopt context-sensitive strategies, and fusion outcomes are strongly influenced by clinicians’ prioritization of prevention, economic incentives and the presence of “prevention champions” [40]. Abdul Raheem analyzed the global status of integrating preventive levels into clinical practice, identifying excessive resource allocation toward disease management, low coverage of preventive services, and barriers such as time constraints and insufficient awareness [41]. Gizaw through a systematic review, identified ten key strategies—including community health programs, school health services, and mobile clinics—that enhance accessibility and resource integration in rural settings by addressing geographic barriers and resource shortages [42]. And syntheses on community health workers document their role in bridging marginalised populations to services, addressing social determinants and enhancing chronic-disease control and cost-effectiveness [43].

### 2.4. Research gaps and the positioning of this study

Current academic research on this integration faces several limitations: Focus: Studies primarily emphasize coordination between hospitals and specialized public health agencies, leaving the internal integration within PHIs under-researched. Methodology: Research is largely dominated by qualitative analysis and mechanism exploration, lacking quantitative models to dynamically evaluate coordination levels and diagnose obstacles between the two systems. Granularity: PHIs are often treated as a homogenous group, overlooking differences in functional positioning and resource allocation between township health centers and community health service centers. Temporal Scope: A lack of long-term longitudinal tracking makes it difficult to reveal the dynamic evolution and shifting trends of barrier factors. Addressing these gaps, this paper innovatively introduces the Coupling Coordination Degree (CCD) model, Relative Development model, and Obstacle Degree model into the study of primary-level medical-preventive integration. By utilizing the Entropy Weight Method, we achieve a dynamic quantitative evaluation and diagnostic analysis of the synergistic development between medical and public health services. The contributions of this study are fourfold: Quantitative Breakthrough: It shifts the focus from external coordination to internal system integration, filling a void in quantitative empirical research within PHIs. Comprehensive Indicators & Longitudinal Analysis: By constructing a comprehensive index system covering medical services, public health services, and health resources, and utilizing panel data from 2017 to 2024, this study reveals the spatio-temporal evolution, periodic disturbances, and policy lag effects in Hebei Province. Heterogeneity Analysis: By distinguishing between township health centers and community health service centers, the study identifies a “dual-track” development pattern where township centers significantly outperform community centers. Precision Diagnosis: Through obstacle degree analysis, the research shifts from result evaluation to process diagnosis, uncovering the underlying logic of the medical-preventive disconnect. These findings provide a precise empirical basis for categorized policymaking and offer actionable references for high-quality integration in Hebei Province and similar regions across China.

## 3. Materials and methods

### 3.1. Research subjects and data sources

The research subjects of this study encompass all township health centers and community health service centers across Hebei Province. The research data were extracted from the Hebei Provincial Health Statistical Yearbook (2018–2025), as detailed in Table 1.

**Table 1:** The number of township health centers and community health service centers in Hebei Province from 2017 to 2024.

Institutional Classification	2017	2018	2019	2020	2021	2022	2023	2024
Township Health Centers	1980	2006	1998	1998	1970	1970	1965	1965
Community Health Service Centers	287	326	343	340	342	345	364	407

### 3.2. Research Methods

#### 3.2.1. Construction and Authority Validation of the Indicator System.

The scientific rigor of the evaluation indicator system serves as the foundation for empirical analysis. Drawing upon the theoretical frameworks established by scholars such as Yuan Beibei [44], Guo Jia [45]and Li Cancan [46]^,^ this study preliminarily constructed an indicator system for the coupling and coordinated development of medical and public health services in primary healthcare institutions (PHIs). To ensure the authority and applicability of this system within the field of primary health management, a modified Delphi method was employed for validation and optimization.

During the validation process, a panel of 21 experts was assembled, comprising representatives from Health Commissions, Centers for Disease Control and Prevention (CDCs), medical universities, and lead administrators of high-performing PHIs across Hebei Province and the Beijing-Tianjin region. The expert selection criteria were strictly defined as follows: a minimum of 10 years of professional experience in relevant fields, holding a senior professional title, or serving in an administrative position at the division level or above. The structured description of its professional background is shown in Table 2.

**Table 2:** Composition of Delphi Experts.

Dimension	Category	No. of Experts	Proportion	Description
Professional background	Clinical medicine experts	7	33.30%	Mainly from hospitals and primary medical institutions
	Public health / CDC experts	7	33.30%	Mainly from centers for disease control and public health institutions
	Health management / administrative experts	7	33.30%	Mainly from health commissions, universities, and administrative departments
Unit type	Health administrative departments	5	23.80%	Provincial, municipal, and county-level health commission systems
	Centers for Disease Control and Prevention	6	28.60%	Provincial and municipal CDC institutions
	Higher medical institutions	5	23.80%	Medical schools and schools of public health
	Primary medical and health institutions	5	23.80%	Township health centers and community health service centers
Title level	Senior professional title	9	42.90%	Professor, chief physician, chief technician, etc.
	Associate senior professional title	10	47.60%	Associate professor, deputy chief physician, deputy chief technician, etc.
	Administrative position	2	9.50%	Division-level position or above
Years of work experience	10–14 years	4	19.00%	Meets inclusion criteria
	15–19 years	6	28.60%	Meets inclusion criteria
	20 years and above	11	52.40%	Meets inclusion criteria

The assessment of indicator validity centers on the calculation of the expert authority coefficient ${\text{C}}_{\text{r}}$, which is jointly determined by the experts’ judgment basis ${\text{C}}_{\text{a}}$ and their degree of familiarity with the indicators The formula for its calculation is as follows:


$$\begin{array}{c} {\text{C}}_{\text{r}}=\frac{{\text{C}}_{\text{a}}+{\text{C}}_{\text{s}}}{\text{2}} \end{array}$$

Based on the results of the two rounds of expert consultation, the mean value of experts’ familiarity with the indicator system was ${\text{C}}_{\text{s}}=\text{0}.\text{94}$, and the mean value of the judgment basis was ${\text{C}}_{\text{a}}=\text{0}.\text{88}$. The calculated expert authority coefficient was therefore Cr = 0.91. According to established academic standards, a value of ${\text{C}}_{\text{r}}\geq$0.7 indicates a relatively high level of authority and reliability of the research findings. The ${\text{C}}_{\text{r}}$ obtained in this study substantially exceeds this threshold, thereby demonstrating that the constructed indicator system can accurately capture the connotation of the integration of medical care and prevention at the primary level.

In addition, the questionnaire response rates for both rounds of consultation reached 100%. The Kendall’s coefficient of concordance was statistically significant P < 0.001 indicating a high degree of consensus among the experts. These results confirm the strong academic rigor and practical relevance of the established evaluation index system.

The finalized indicator system comprises two major categories: medical services and public health services. Each category encompasses two core dimensions, namely health resources and service provision. Under the health resources dimension, expenditure-related indicators were uniformly selected, with one indicator included for medical services and one for public health services. Under the service provision dimension, indicators reflecting service volume and efficiency were incorporated, including eight indicators for medical services and seventeen indicators for public health services. Detailed information is presented in Table 3.

**Table 3:** Index System for the Coupling and Coordinated Development of Medical Services and Public Health Services in Primary Medical and Health Institutions.

Service Type	Primary Indicators	Secondary Indicators	Polarity	Unit	Code	Weight
Medical Services	Health Resources	Medical Expenditure	+	1,000 RMB	M1	0.119
	Service Provision	Average Daily Outpatient Visits per Physician	+	Visits	M2	0.065
		Average Daily Inpatient Bed-days per Physician	+	Days	M3	0.230
		Total Number of Outpatient and Emergency Visits	+	Visits	M4	0.137
		Total Number of Admissions	+	Persons	M5	0.223
		Number of Home Health Service Visits	+	Visits	M6	0.178
		Bed Utilization Rate	+	%	M7	0.043
		Average Length of Stay	–	Days	M8	0.006
Public Health Services	Health Resources	Public Health Expenditure	+	1,000 RMB	P1	0.057
	Service Provision	Number of Health Examinations	+	Visits	P2	0.053
		Cumulative Number of Health Records Established	+	Persons	P3	0.059
		Number of Standardized Electronic Health Records	+	Persons	P4	0.058
		Total Beneficiaries of Public Health Consultation Activities	+	Persons	P5	0.074
		Total Beneficiaries of Health Education Lectures	+	Persons	P6	0.077
		Vaccinations under National Immunization Program (0–6 years)	+	Persons	P7	0.054
		Number of Children under Health Management (0–6 years)	+	Persons	P8	0.062
		Number of Maternal Health Management Cases	+	Persons	P9	0.066
		Number of Seniors under Health Management (65 + years)	+	Persons	P10	0.057
		Standardized Management of Patients with Hypertension	+	Persons	P11	0.065
		Standardized Management of Patients with Diabetes	+	Persons	P12	0.063
		Standardized Management of Patients with Severe Mental Disorders	+	Persons	P13	0.059
		Health Management of Patients with Pulmonary Tuberculosis	+	Persons	P14	0.069
		Reported Cases of Infectious Diseases and PHEs*	–	Cases	P15	0.004
		Number of Health Inspection and Coordination Patrols	+	Times	P16	0.067
		Number of People under TCM Health Management	+	Persons	P17	0.057

#### 3.2.2. Determination of indicator weights.

①Data Standardization To eliminate the influence of different dimensions and scales on the subsequent entropy weight calculations, this study employs the Min-Max Normalization Range Method to standardize the raw data. The formulas are as follows:


$$\begin{array}{c} \text{For positive indicators}:\;{\text{x}}_{\text{ij}}=\frac{\overset{\prime }{\underset{\text{ij}}{\text{x}}}-\text{min}(\overset{\prime }{\underset{\text{j}}{\text{x}}})}{\text{max}\left(\overset{\prime }{\underset{\text{j}}{\text{x}}}\right)-\text{min}(\overset{\prime }{\underset{\text{j}}{\text{x}}})}+\text{0}.\text{0001} \end{array}$$

$$\begin{array}{c} \text{For negative indicators}:\;{\text{x}}_{\text{ij}}=\frac{\text{max}\left(\overset{\prime }{\underset{\text{j}}{\text{x}}}\right)-\overset{\prime }{\underset{\text{ij}}{\text{x}}}}{\text{max}\left(\overset{\prime }{\underset{\text{j}}{\text{x}}}\right)-\text{min}(\overset{\prime }{\underset{\text{j}}{\text{x}}})}+\text{0}.\text{0001} \end{array}$$

In these equations, ${\text{X}}_{\text{ij}}$ represents the raw value of the j-th indicator for the i-th year (or unit), while ${\text{X}}_{\text{ij}}$ denotes the standardized value. $\text{max}({\text{X}}_{\text{j}})$ and $\text{min}({\text{X}}_{\text{j}})$ are the maximum and minimum values of the j-th indicator across all observation years, respectively. Since the Entropy Weight Method utilizes logarithmic functions to determine information entropy, standardized values of zero lead to mathematical undefinedness, undermining the integrity of the weight determination process. To address this limitation, a translation adjustment is applied by incorporating a small constant (0.0001) during standardization, such that all values exceed zero. This approach facilitates computational feasibility and numerical stability without distorting the underlying relative variances or the hierarchical order of the dataset. Consequently, it bolsters the reliability and robustness of the derived weights.

②Weight Calculation using the Entropy Weight Method The weights were determined using the entropy weight method, which minimizes subjective bias by assigning weights based on the objective information provided by the data. The calculation steps are as follows:


$$\begin{array}{c} \text{Contribution}:\;{\text{p}}_{\text{ij}}=\frac{{\text{x}}_{\text{ij}}}{\sum_{\text{i}=\text{1}}^{\text{m}}{\text{x}}_{\text{ij}}} \end{array}$$

$$\begin{array}{c} \text{Entropy Value}:\;{\text{e}}_{\text{j}}=\frac{\sum_{\text{i}=\text{1}}^{\text{m}}{\text{p}}_{\text{ij}}\text{ln}{\text{p}}_{\text{ij}}}{\text{ln}\text{m}} \end{array}$$

$$\begin{array}{c} \text{Coefficient of Variation}:\;\;{\text{d}}_{\text{j}}=\text{1}-{\text{e}}_{\text{j}} \end{array}$$

$$\begin{array}{c} \text{Indicator Weight}:\;{\text{w}}_{\text{j}}=\frac{{\text{d}}_{\text{j}}}{\sum_{\text{i}=\text{1}}^{\text{n}}{\text{d}}_{\text{j}}} \end{array}$$

In these formulas, ${\text{P}}_{\text{ij}}$ represents the contribution of the j-th indicator in the i-th sample, where m is the number of samples; ${\text{e}}_{\text{j}}$ denotes the entropy value of the j-th indicator; ${\text{g}}_{\text{j}}$ represents the coefficient of variation for the j-th indicator; and ${\text{W}}_{\text{j}}$ is the final weight of the j-th indicator, where n is the total number of indicators.

#### 3.2.3. Analysis of coupling and coordinated development.

Based on the Capacity Coupling Theory, a system synergy analysis model was constructed to reveal the coordinative relationship between the two systems: medical services and public health services at the primary level. The primary steps are as follows [47]:

①Calculation of Composite Evaluation Scores The composite scores for the two evaluation systems are calculated using the following formulas:


$$\begin{array}{c} \;{\text{U}}_{\text{1},\text{2}}=\sum_{\text{j}=\text{1}}^{\text{n}}{\text{w}}_{\text{j}}\times {\text{x}}_{\text{ij}} \end{array}$$

Here, ${\text{U}}_{\text{1}}$ denotes the composite score of the medical service subsystem, and ${\text{U}}_{\text{2}}$ denotes the composite score of the public health service subsystem.

②The Coupling Coordination Degree (CCD) model is calculated using the following formulas:


$$\begin{array}{c} \text{C}=\frac{\text{2}\sqrt{\text{U1}\times \text{U2}}}{\text{U1}+\text{U2}} \end{array}$$

$$\begin{array}{c} \text{T}=\alpha \text{U1}+\beta \text{U2}(\alpha =\beta =\text{0}.\text{5}) \end{array}$$

$$\begin{array}{c} \text{D}=\sqrt{\text{C}\times \text{T}} \end{array}$$

The coupling degree (C) measures the extent of interaction and coordination between the two subsystems. A higher value of (C) indicates a stronger level of interdependence and mutual influence. According to its magnitude, the coupling process can be classified into four stages: low-level coupling, antagonistic stage, running-in stage, and high-level coupling. The comprehensive coordination index (T) reflects the overall level of development of the two subsystems. The parameters α and β are weighting coefficients subject to the constraint α + β = 1. Given that medical services and public health services are considered to be of equal importance in this study, the weights are set as α = β = 0.5. The coupling–coordination degree (D) provides an integrated assessment of the overall coordination level between the two subsystems. Its value ranges from 0 to 1 and is categorized into ten grades in ascending order: extreme maladjustment, severe maladjustment, moderate maladjustment, mild maladjustment, on the verge of maladjustment, barely coordinated, primary coordination, intermediate coordination, good coordination, and high-quality coordination.

#### 3.2.4. Relative development model.

While the Coupling Coordination Degree (CCD) can effectively assess the level of synergy between systems, it is insufficient for capturing the dynamic matching degree between supply and demand. Consequently, a Relative Development Model was established to measure the relative developmental status of the two systems. The formula is as follows:


$$\begin{array}{c} \text{R}=\frac{{\text{U}}_{\text{1i}}}{\sum_{\text{i}=\text{1}}^{\text{n}}{\text{U}}_{\text{1i}}}/\frac{{\text{U}}_{\text{2i}}}{\sum_{\text{i}=\text{1}}^{\text{n}}{\text{U}}_{\text{2i}}} \end{array}$$

Here, R denotes the relative development degree; ${\text{U}}_{\text{1i}}$ represents the medical service score of the i-th sample; and ${\text{U}}_{\text{2i}}$ represents the public health service score of the i-th sample. Following prevailing criteria in coupling coordination literature, 0.8 and 1.2 are set as the empirical thresholds for relative development [48,49]. Since 1 represents the equilibrium benchmark, the 0.8–1.2 interval is considered a tolerance zone for reasonable fluctuations, accounting for measurement biases and routine systemic discrepancies. Consequently, this interval is used to assess the synchronicity of subsystems; values outside this range signify a pronounced relative lag in one of the systems. When R≤0.8, the pattern is classified as a medical-service-lagging type. When 0.8≤R≤1.2, it is categorized as a balanced development type between medical services and public health services. When R≥1.2, it is identified as a public-health-service-lagging type.

#### 3.2.5. Obstacle degree function model.

To more accurately identify the key factors constraining the synergistic evolution of the two systems, an Obstacle Factor Diagnostic Model was employed to provide an in-depth analysis of the hindering factors. The primary formulas are as follows [50]:


$$\begin{array}{c} {\text{F}}_{\text{i}}={\text{w}}_{\text{j}}\times {\text{a}}_{\text{ij}} \end{array}$$

$$\begin{array}{c} \text{Ii}=\text{1}-{\text{X}}_{\text{i}} \end{array}$$

$$\begin{array}{c} {\text{H}}_{\text{j}}=\frac{{\text{F}}_{\text{i}}\times {\text{l}}_{\text{i}}}{\sum_{\text{i}=\text{1}}^{\text{n}}{\text{F}}_{\text{i}}\times {\text{l}}_{\text{i}}}\times \text{1}\text{00}\% \end{array}$$

Fi denotes the factor contribution degree, representing the importance of the i-th indicator to the coupling–coordination level of the binary composite system. ${\text{w}}_{\text{j}}$denotes the weight of the j-th subsystem within the composite system. ${\text{a}}_{\text{ij}}$ represents the weight of the i-th indicator in the j-th subsystem. The indicator deviation degree, Ii, reflects the extent to which the actual value of the i-th indicator deviates from its optimal value. ${\text{X}}_{\text{i}}$ denotes the standardized value of the i-th indicator. ${\text{H}}_{\text{j}}$ represents the obstacle degree of the j-th indicator. A larger obstacle degree indicates a stronger inhibitory effect of the corresponding indicator on the coupling–coordination level, whereas a smaller value suggests a weaker constraining effect.

## 4. Results

### 4.1. Composite development levels of medical and public health services in PHIs

From 2017 to 2024, the composite development levels of medical and public health services in Hebei Province’s primary healthcare institutions (PHIs) generally remained within a moderate range, characterized by significant periodic fluctuations and asynchronous evolutionary features. Specifically, the composite level of medical services was slightly higher than that of public health services, stabilizing around 0.50 in most years. However, this indicator experienced a marked decline during the 2019–2020 period, reaching a nadir of approximately 0.38, before gradually recovering to a relative peak of approximately 0.53 in 2023–2024. In contrast, the composite development level of public health services remained stable within the 0.44–0.48 range, exhibiting smaller inter-annual fluctuations.

Analysis by institutional type reveals that Township Health Centers (THCs) significantly outperformed Community Health Service Centers (CHSCs) in the composite development levels of both service categories, with more pronounced inter-annual fluctuations. Specifically: Township Health Centers: The composite scores for medical services ranged from 0.131 to 0.633, while public health services ranged from 0.026 to 0.327. Community Health Service Centers: Development levels for both services remained low. Medical service scores were below 0.30 in most years, and public health scores fluctuated between 0.009 and 0.163, with indicators in cities like Hengshui and Chengde approaching zero. In summary, THCs maintain a relative advantage in both service domains—particularly in public health—though they are subject to greater volatility. Conversely, CHSCs operate at a consistently low level, with the lag in public health service development being particularly prominent.

### 4.2. Coupling degree and comprehensive coordination index of medical and public health services in PHIs

The research results indicate that the medical and public health services of primary healthcare institutions (PHIs) in Hebei Province are in a high-level coupling stage. From 2017 to 2024, the medical-preventive coupling degree ($C$) remained consistently stable at an extremely high level above 0.995, reaching 1.000 in both 2017 and 2019. This suggests that the institutional linkages and macro-coordination frameworks for primary-level medical-preventive integration have formed a close and stable dynamic mechanism. At the institutional level: Township Health Centers (THCs): The coupling degree has been maintained within a high-level range of 0.950–1.000 since 2017, with minimal inter-annual fluctuations, reflecting strong stability in the synergistic linkage between medical and public health services. Community Health Service Centers (CHSCs): The coupling degree showed a significant upward trend, with most municipalities stabilizing around 0.900 in recent years. However, regional disparities remain stark; for instance, the coupling degree for CHSCs in Chengde City has long languished at a low level of 0.35–0.48, highlighting pronounced regional heterogeneity in the advancement of medical-preventive integration across Hebei Province.

From 2017 to 2024, the Comprehensive Coordination Index of medical-preventive integration in Hebei’s PHIs exhibited significant hierarchical stratification and regional differences. The provincial index remained within a relatively low range (fluctuating between 0.212 and 0.249), showing only a slow and marginal upward trend. The differentiation by institutional type is particularly prominent, with the coordination level of Township Health Centers (X) significantly outperforming that of Community Health Service Centers (S). THCs: Overall coordination reached a medium-to-high level, with most municipalities and years stabilizing between 0.38 and 0.62. Regional performance varied: Handan and Baoding consistently led the province, Shijiazhuang showed a fluctuating recovery trend, while Qinhuangdao, Langfang, and Hengshui remained at low levels for a prolonged period. CHSCs: The coordination index remained trapped in a low range of 0.03–0.15. Although indicators in some municipalities rose above 0.20 by 2024, the overall improvement was limited. The vast gap between CHSCs and THCs further underscores the imbalance in the coordinated development of medical-preventive integration at the primary level in Hebei Province. Detailed data are presented in Table 4.

**Table 4:** Integrated Medical and Preventive Coordination Index of Township/Community Health Institutions in Hebei’s Prefecture-level Cities from 2017 to 2024.

Region	2017		2018		2019		2020		2021		2022		2023		2024	
	X	S	X	S	X	S	X	S	X	S	X	S	X	S	X	S
Shijiazhuang	0.542	0.159	0.564	0.168	0.562	0.185	0.566	0.162	0.478	0.140	0.477	0.163	0.545	0.225	0.578	0.245
Tangshan	0.411	0.118	0.430	0.117	0.430	0.138	0.391	0.132	0.416	0.136	0.379	0.138	0.433	0.160	0.437	0.171
Qinhuangdao	0.213	0.042	0.254	0.050	0.231	0.053	0.190	0.069	0.186	0.071	0.201	0.040	0.265	0.055	0.262	0.079
Handan	0.653	0.080	0.637	0.063	0.577	0.075	0.561	0.065	0.545	0.057	0.503	0.112	0.562	0.097	0.599	0.094
Xingtai	0.477	0.063	0.458	0.055	0.412	0.048	0.381	0.054	0.390	0.057	0.445	0.061	0.532	0.067	0.551	0.078
Baoding	0.629	0.118	0.618	0.143	0.564	0.124	0.524	0.101	0.426	0.075	0.403	0.084	0.459	0.101	0.493	0.123
Zhangjiakou	0.287	0.104	0.315	0.115	0.278	0.113	0.252	0.067	0.241	0.066	0.239	0.065	0.258	0.088	0.254	0.079
Chengde	0.349	0.067	0.341	0.147	0.307	0.154	0.276	0.150	0.295	0.136	0.302	0.116	0.319	0.124	0.307	0.125
Cangzhou	0.395	0.092	0.393	0.086	0.386	0.079	0.364	0.067	0.372	0.056	0.374	0.036	0.379	0.048	0.396	0.048
Langfang	0.282	0.061	0.236	0.056	0.214	0.063	0.200	0.031	0.201	0.042	0.202	0.058	0.224	0.071	0.251	0.082
Hengshui	0.239	0.021	0.250	0.035	0.231	0.032	0.208	0.031	0.218	0.031	0.217	0.035	0.230	0.034	0.249	0.074

Note: X represents Township Health Centers; S represents Community Health Service Centers.

To examine the influence of weight settings in the composite coordination index (T) on the research findings, a sensitivity analysis was conducted. Specifically, α was alternatively set to 0.4 and 0.6, and the coupling coordination degree (D) for each region was recalculated accordingly. Notably, the coordination performance of township health centers consistently exceeded that of community health centers, thereby confirming that the primary conclusions concerning the divergent development of these primary healthcare institutions are highly robust. See “S1 Table and S2 Table” for details.

### 4.3. Coupling coordination degree of medical and public health services in PHIs

The coupling coordination level of medical-preventive integration in Hebei Province’s primary healthcare institutions (PHIs) is characterized by significant institutional differentiation, with the coupling coordination degree (D) of Township Health Centers (THCs) being generally higher and possessing superior coordination grades compared to community health service centers (CHSCs). From an overall perspective, the D values for THCs primarily fluctuated within a medium-to-high range, with annual values distributed between 0.454 and 0.808, and the dominant coordination categories were “barely coordinated” and “intermediate coordination”. In contrast, the coupling coordination degree for CHSCs was significantly lower, ranging from 0.137 to 0.481, with coordination types concentrated in low-level categories such as “mild disorder” and “on the verge of disorder”; notably, the D values for such institutions in Hengshui City remained in the “serious disorder” range (0.1 ＜ D ≤ 0.2) for a prolonged period. Analysis of temporal evolution shows that the coupling coordination degree of THCs remained stable overall, with some municipalities exhibiting a marginal recovery trend since 2022; although CHSCs in certain cities demonstrated gradual improvements, the overall magnitude of progress was limited, and regional disparities remained prominent, with the coordination level still lagging significantly behind that of THCs. See Table 5.

**Table 5:** Coupling Coordination Degree of Medical and Preventive Services of Township Health Centers/Community Health Service Centers in Various Prefecture-level Cities of Hebei Province from 2017 to 2024.

Region	2017		2018		2019		2020		2021		2022		2023		2024	
	X	S	X	S	X	S	X	S	X	S	X	S	X	S	X	S
Shijiazhuang	0.730	0.386	0.747	0.399	0.746	0.422	0.746	0.398	0.679	0.373	0.684	0.401	0.738	0.463	0.761	0.481
Tangshan	0.638	0.292	0.654	0.300	0.654	0.328	0.620	0.321	0.637	0.329	0.610	0.335	0.658	0.362	0.661	0.378
Qinhuangdao	0.454	0.200	0.489	0.213	0.470	0.220	0.435	0.246	0.431	0.248	0.443	0.199	0.495	0.230	0.490	0.262
Handan	0.808	0.234	0.798	0.215	0.759	0.268	0.748	0.251	0.735	0.240	0.702	0.334	0.749	0.306	0.773	0.305
Xingtai	0.690	0.250	0.676	0.235	0.640	0.219	0.612	0.231	0.619	0.238	0.663	0.247	0.724	0.258	0.738	0.278
Baoding	0.784	0.337	0.775	0.358	0.734	0.342	0.683	0.318	0.623	0.274	0.608	0.290	0.669	0.316	0.699	0.343
Zhangjiakou	0.530	0.274	0.554	0.289	0.523	0.300	0.500	0.242	0.491	0.241	0.488	0.238	0.504	0.267	0.503	0.269
Chengde	0.579	0.207	0.573	0.253	0.548	0.233	0.523	0.230	0.540	0.217	0.544	0.217	0.553	0.218	0.544	0.246
Cangzhou	0.596	0.262	0.591	0.261	0.584	0.257	0.560	0.245	0.566	0.235	0.569	0.188	0.587	0.219	0.609	0.216
Langfang	0.531	0.185	0.486	0.194	0.457	0.227	0.433	0.174	0.436	0.200	0.437	0.229	0.468	0.258	0.500	0.275
Hengshui	0.485	0.137	0.498	0.174	0.476	0.179	0.444	0.177	0.456	0.174	0.455	0.183	0.476	0.182	0.497	0.273

Note: X represents Township Health Centers; S represents Community Health Service Centers.

### 4.4. Relative Development Degree of Medical Services and Public Health Services in Primary Medical and Health Institutions

From 2017 to 2024, significant imbalances and pronounced regional disparities were observed in the relative development of medical services and public health services among primary medical and health institutions in Hebei Province. Between 2017 and 2019, township health centers were predominantly characterized by a medical-service-lagging pattern R ≤ 0.8. By 2024, most prefecture-level cities had shifted toward a balanced development pattern 0.8 ≤ R ≤ 1.2. However, cities such as Qinhuangdao and Chengde remained in a public-health-service-lagging state R > 1.2 over an extended period, exhibiting a structural asymmetry in which medical services were relatively more advanced. In contrast, community health service centers were generally characterized by a public-health-service-lagging pattern, with most cities maintaining R ≥ 1.2 throughout the study period. In particular, Chengde and Tangshan displayed extremely elevated values with Chengde exceeding 15 for multiple years and Tangshan consistently above 3, indicating a persistent relative lag in public health services and an evident imbalance in the integrated development of medical care and prevention. See Table 6 for details.

**Table 6:** Relative Development Degree of Medical and Preventive Integration of Township Health Centers/Community Health Service Centers in Various Prefecture-level Cities of Hebei Province from 2017 to 2024.

Region	2017		2018		2019		2020		2021		2022		2023		2024	
	X	S	X	S	X	S	X	S	X	S	X	S	X	S	X	S
Shijiazhuang	0.69	2.08	0.77	1.93	0.74	1.77	0.69	1.55	0.58	1.32	0.67	1.41	1.00	1.86	1.02	2.00
Tangshan	0.78	5.42	0.81	4.52	0.80	4.31	0.70	4.34	0.65	4.08	0.67	3.74	1.02	3.69	1.01	3.45
Qinhuangdao	1.67	1.93	2.02	2.50	1.83	2.35	1.28	2.92	1.23	2.98	1.53	1.01	2.22	1.69	2.34	2.87
Handan	0.90	6.43	0.94	5.34	0.88	1.75	0.85	1.58	0.75	1.05	0.65	0.81	0.94	1.75	1.11	1.36
Xingtai	0.89	1.45	0.94	0.96	0.78	0.84	0.69	0.75	0.69	0.89	0.73	0.94	1.42	1.00	1.35	1.37
Baoding	0.66	1.78	0.62	2.66	0.54	1.98	0.38	1.13	0.42	1.10	0.43	1.08	0.64	1.45	0.76	1.82
Zhangjiakou	1.48	5.58	1.61	5.35	1.43	4.09	1.23	2.78	1.09	2.77	1.12	2.84	1.36	3.81	1.21	2.38
Chengde	1.78	7.65	1.77	18.95	1.49	30.24	1.28	29.92	1.33	30.85	1.45	22.15	1.80	25.32	1.75	15.16
Cangzhou	0.39	4.97	0.37	4.09	0.36	3.43	0.33	2.55	0.32	1.38	0.33	0.70	0.42	0.87	0.48	0.58
Langfang	0.93	10.63	0.93	6.82	0.63	3.66	0.49	0.76	0.51	1.75	0.51	2.46	0.67	1.99	0.82	2.28
Hengshui	0.71	2.70	0.79	2.83	0.67	0.95	0.51	1.07	0.53	1.60	0.54	1.94	0.72	1.66	0.80	1.02

Note: X represents Township Health Centers; S represents Community Health Service Centers.

### 4.5. Major obstacle factors affecting the integration of medical and public health services in primary medical and health institutions

An analysis of the principal obstacle factors affecting the integration of medical care and prevention in Hebei Province indicates that, from 2017 to 2024, township health centers (X) and community health service centers (S) exhibited both overlapping and distinctly differentiated core constraints. For township health centers, the number of person-times of family health services (M6) and the number of hospital admissions (M5) consistently ranked among the leading obstacle factors. The dominant constraint demonstrated a phased shift over time. Between 2017 and 2020, the number of family health service person-times constituted the primary obstacle, with obstacle degrees ranging from 8.6% to 9.8%. Beginning in 2021, the number of hospital admissions rose to become the foremost obstacle factor, with obstacle degrees maintained between 7.8% and 8.7%. Meanwhile, the obstacle degree of the average daily inpatient bed-days borne per physician (M3) has increased markedly in recent years, stabilizing within the range of 7.4% to 8.3%. In contrast, community health service centers have consistently identified hospital admissions as the primary long-term obstacle factor. In addition, several public health–related indicators—including the total number of beneficiaries of health education lectures (P6), the total number of beneficiaries of public health consultation activities (P5), and the number of tuberculosis patients under health management (P14)—have repeatedly ranked among the top constraints, with obstacle degrees largely concentrated between 5.0% and 6.0%. The obstacle degree associated with the average daily inpatient bed-days per physician has also exhibited an upward trend. See Table 7 for details.

**Table 7:** Main Obstacle Factors and Obstacle Degrees (%) of Medical and Preventive Integration in Township Health Centers/Community Health Service Centers of Hebei Province from 2017 to 2024.

Ranking	2017		2018		2019		2020		2021		2022		2023		2024	
	X	S	X	S	X	S	X	S	X	S	X	S	X	S	X	S
1	M6 (9.8)	M5 (7.0)	M6 (9.1)	M5 (7.1)	M6 (8.6)	M5 (7.1)	M6 (8.6)	M5 (7.1)	M5 (8.7)	M5 (7.1)	M5 (8.8)	M5 (7.2)	M5 (7.8)	M5 (7.2)	M5 (7.8)	M5 (7.3)
2	P14 (6.9)	P6 (5.7)	M5 (6.7)	P6 (5.7)	M5 (7.5)	P6 (5.8)	M5 (8.1)	P6 (5.7)	M3 (8.1)	M3 (6.0)	M3 (8.3)	M3 (6.1)	M3 (7.4)	M3 (6.0)	P5 (7.5)	M3 (6.1)
3	P16 (6.7)	M6 (5.6)	P14 (6.6)	M6 (5.7)	P14 (6.4)	M6 (5.6)	M3 (7.2)	M3 (5.6)	M6 (6.9)	P6 (5.7)	M6 (6.3)	P6 (5.7)	P14 (6.7)	P6 (5.8)	M3 (7.4)	P6 (5.8)
4	M5 (6.6)	M3 (5.3)	P6 (6.6)	P5 (5.4)	P6 (6.2)	P5 (5.4)	P14 (6.5)	M6 (5.6)	P14 (6.3)	M6 (5.5)	P5 (5.9)	M6 (5.4)	P5 (6.6)	P5 (5.5)	P9 (6.3)	P5 (5.6)
5	P6 (6.5)	P5 (5.3)	P16 (6.1)	P14 (5.1)	M3 (6.1)	M3 (5.1)	P5 (5.7)	P5 (5.4)	P5 (5.7)	P5 (5.4)	P14 (5.9)	P5 (5.4)	P9 (6.2)	M6 (5.3)	P14 (6.2)	M6 (5.2)
6	M4 (5.7)	P14 (5.1)	M4 (5.8)	P16 (4.9)	M4 (5.6)	P14 (5.1)	M4 (5.5)	P14 (5.1)	P9 (5.4)	P14 (5.0)	P9 (5.7)	P14 (5.1)	P6 (6.0)	P14 (5.1)	P6 (5.6)	P14 (5.0)

Note: X represents Township Health Centers; S represents Community Health Service Centers.

## 5. Discussion

### 5.1. The underlying structural contradiction of high coupling but low coordination in primary medical–preventive integration

The findings indicate that from 2017 to 2024, the coupling degree of medical and public health services in primary health institutions in Hebei Province remained at a relatively high level, whereas the coordination index persistently fluctuated at a low level, exhibiting a typical pattern of “high coupling but low coordination”. This suggests that, at the macro level of policy framework and institutional design, medical services and public health services have established formal systemic linkages aligned with the strategic deployment of the Healthy China initiative. However, at the micro-operational level, the actual effectiveness of integration and synergistic functioning falls substantially short of expectations, resulting in a slow improvement in overall development performance. The root cause of this phenomenon lies in the long-standing operational inertia characterized by “emphasizing treatment over prevention” with misaligned financing and incentive mechanisms constituting the central structural constraint [4]. From the perspective of revenue composition, primary healthcare institutions rely heavily on medical insurance reimbursements derived from curative services, the proportion of which significantly exceeds that of public health subsidies, which are typically project-based and exhibit lag effects. In this study, treatment-related indicators—such as the number of hospital admissions (M5) and the person-times of family health services (M6)—consistently ranked among the principal obstacle factors. This reflects the rational behavioral response of primary institutions under the prevailing incentive structure: increasing clinical service volume serves as a direct and immediate pathway to revenue growth. In contrast, the outcomes of public health services are inherently long-term and characterized by positive externalities. Given their relatively low weight in performance appraisal systems, the motivation of healthcare personnel to engage in preventive and health management activities is correspondingly weakened. This structural imbalance ultimately leads to the persistent lag of public health services behind medical services in terms of comprehensive development.

To address this systemic issue, optimization strategies must transcend superficial coordination and instead focus on reforming the underlying incentive mechanisms. It is recommended that the Provincial Health Commission take the lead, in coordination with medical insurance and financial authorities, to refine the top-level design and supporting policies for medical–preventive integration. First, financing and payment mechanisms should be restructured. On the basis of global budgeting, capitation-based bundled payment models may be explored, alongside the establishment of a dedicated performance pool for medical–preventive integration. Core public health outcome indicators—such as standardized chronic disease management rates, early detection rates of infectious diseases, and improvements in residents’ health literacy—should be closely linked to institutional revenue allocation and healthcare personnel remuneration [51]^.^ Second, a closed-loop evaluation system oriented toward health outcomes should be constructed, guiding primary institutions to shift from a quantity-driven service model to an outcome-focused performance paradigm [52]. Third, provincial-level data empowerment should be strengthened. Leveraging the advantages of public data resources, barriers between basic medical and public health information systems should be dismantled to establish a province-wide, life-course health record system for residents. Through dynamic monitoring and evaluation of the actual health outputs of primary medical–preventive integration, robust data support can be provided for precise incentive design and accountability mechanisms [53].

### 5.2. Township health centers and community health service centers exhibit a pronounced dual development pattern

The study demonstrates that Township Health Centers outperform community health service centers in terms of the overall level of medical–preventive integration and coordination. This “dual development” pattern reveals substantial internal heterogeneity within primary healthcare institutions—a critical characteristic that has not received sufficient attention in prior research. The emergence of this pattern cannot be attributed to a single factor; rather, it is the result of the combined influence of multidimensional structural conditions. At the level of functional positioning and resource allocation, Township Health Centers generally possess inpatient service capacity. Their bed supply and staffing structures are more compatible with rural population density and healthcare demand, enabling them to naturally integrate diagnosis, treatment, prevention, follow-up, and referral into a continuous service chain for common diseases, chronic conditions, and infectious disease control. In contrast, urban community health service centers, although functionally oriented toward health management and basic medical care, are constrained by the absence of inpatient beds and limited talent attraction capacity. Moreover, the high concentration of high-quality medical resources in urban tertiary hospitals has substantially crowded out the first-contact function of community institutions, weakening their role in primary diagnosis and trapping them in a state of marginalization in clinical service provision. Consequently, they struggle to serve as reliable hubs for residents’ health management. Simultaneously, preventive services in community health service centers often lack solid clinical support, resulting in a more pronounced disjunction between medical and preventive services compared with Township Health Centers. From a governance perspective, Township Health Centers are typically positioned as key nodes within tightly integrated county-level medical consortia spanning county–township–village networks. This positioning facilitates the downward transfer of resources and coordinated policy support at the county level. By contrast, urban community health service centers operate within multi-departmental and multi-tiered administrative systems, where cross-sectoral coordination and integrated reform face comparatively greater institutional complexity.

In light of these developmental disparities, optimization strategies for medical–preventive integration should adhere to the principles of differentiated policy design and targeted empowerment. For Township Health Centers, it is essential to further consolidate their role as foundational nodes within county-level medical consortia. Deepening the construction of tightly integrated county healthcare alliances—through bundled medical insurance payments, pooled establishment quota management, and personnel mechanisms such as “county-managed, township-utilized” staffing—can promote the institutionalized downward flow of high-quality medical resources and technical expertise. This would enable township institutions to comprehensively strengthen their capacity in emergency stabilization, diagnosis and treatment of common and prevalent diseases, implementation of public health programs, and continuous management of chronic conditions within their jurisdictions [11]. For community health service centers, the core objective is to rebuild residents’ trust and service adherence by enhancing substantive service capacity and strengthening vertical coordination mechanisms. On the one hand, infrastructure improvements—such as establishing day-care beds, infusion units, or rehabilitation beds—and institutional innovations—such as permitting specialists from higher-level hospitals to operate community-based studios or conduct regular outreach clinics—can reinforce the clinical foundation of community services and gradually redirect patients with common and chronic diseases back to primary care settings. On the other hand, deeper integration with municipal and district-level hospitals through medical alliances should be pursued, supported by interoperable information systems. A “general practitioner + specialist” collaborative service model may be developed, whereby chronic disease management tasks for conditions such as hypertension and diabetes are clearly delegated to community-based general practitioner teams, while higher-level specialists provide remote consultation, periodic technical guidance, and quality supervision. Such arrangements would facilitate organic integration and efficient coordination across the medical–preventive service continuum [54].

### 5.3. Significant spatiotemporal disparities and stage-specific disturbances in the level of medical–preventive integration

The results indicate substantial intercity disparities in the coupling–coordination level of medical–preventive integration among primary healthcare institutions across Hebei Province. Moreover, the comprehensive coordination index experienced a pronounced decline during 2020–2021. This pattern suggests that the development trajectory of medical–preventive integration is not linear, but rather shaped by the combined effects of external shocks and internal structural adjustments. Regional heterogeneity primarily stems from uneven economic development, urban–rural demographic structures, disparities in health resource allocation, and variations in local governmental implementation capacity. For instance, township health centers in Handan and Baoding have demonstrated relatively strong integration performance, closely associated with the in-depth advancement of tightly integrated county-level medical consortia and the reinforcement of the primary care “gatekeeper” function. In contrast, the long-term lag in community health service centers in cities such as Chengde and Hengshui reveals structural constraints linked to limited local resource endowments and the siphoning effect of concentrated high-quality urban medical resources. The fluctuation observed during 2020–2021 underscores the “double-edged sword” effect of major public health emergencies. On the one hand, the COVID-19 pandemic compelled primary healthcare institutions to strengthen public health emergency response functions, thereby increasing attention to and enforcement of preventive responsibilities. On the other hand, the temporary reallocation of substantial medical and human resources to frontline epidemic control disrupted routine service delivery, exposing deficiencies in systemic resilience and adaptive capacity. It is imperative to clarify, however, that the observed dip in the index during this period does not necessarily equate to a substantive erosion of medical-preventive integration capacity. Amidst the exigencies of pandemic containment, PHIs operated under multifaceted constraints, including the realignment of clinical priorities, an abrupt escalation in public health mandates, personnel mobilization, and systemic latencies in data reporting. Thus, observed indicator volatility likely stems from service delivery fluctuations and reporting lags rather than an intrinsic, unidirectional decline in integration performance. Notably, when epidemic control tasks reached their zenith, the prioritization of emergency response often came at the expense of routine service continuity, highlighting the systemic fragility and constrained elasticity of the integration framework during public health emergencies. Given the absence of control variables such as pandemic intensity, staffing dynamics, and reporting throughput, these findings should be construed as cautious inferences derived from temporal trends rather than rigorous causal identification.

In response to these complex spatiotemporal dynamics, optimization of medical–preventive integration should integrate macro-level coordination with regionally differentiated strategies. At the provincial level, a dynamic monitoring and evaluation feedback mechanism for medical–preventive integration should be established.A periodically released “Primary Medical–Preventive Integration Development Index“ could provide stratified profiling and ranking of cities and institutional types, serving as an evidence base for resource allocation and policy adjustment. For cities and institutional categories exhibiting lagging development, targeted support measures should be implemented, including preferential allocation of public health subsidies, talent recruitment programs, and equipment investment [55].Simultaneously, system resilience must be strengthened by promoting institutional arrangements that integrate emergency response mechanisms with routine services such as chronic disease management and infectious disease surveillance. Establishing a dual-mode operational framework that accommodates both routine and emergency conditions—including flexible personnel deployment plans—would ensure continuity of essential health management services during crises, thereby facilitating the transition of medical–preventive integration from a reactive, crisis-driven model to a stable and sustainable governance paradigm [56].

### 5.4. Conclusion

This study advances the existing literature by moving beyond the prevailing focus on external coordination between hospitals and specialized public health institutions and instead shifting the analytical lens inward to primary healthcare institutions. By centering on intra-organizational integration mechanisms, it not only identifies the core characteristic pattern of “high coupling but low coordination” in Hebei Province and delineates its spatiotemporal evolution, but also challenges the conventional assumption of homogeneity among primary-level institutions. Specifically, it demonstrates a dualistic development structure between township health centers and community health service centers, clarifies their heterogeneous obstacle factors, and provides an in-depth examination of the structural contradictions underlying the fragmentation between medical and preventive services. In doing so, the study offers theoretically grounded and operationally actionable empirical evidence to support the high-quality development of primary-level medical–prevention integration.

Nevertheless, several limitations warrant consideration. First, the empirical scope is confined to Hebei Province; caution is therefore required when generalizing the findings to provinces with markedly different socioeconomic contexts. Second, the study relies primarily on data from official health statistical yearbooks. While authoritative and standardized, these data lack qualitative insights into patient service satisfaction and healthcare workers’ micro-level perceptions, making it difficult to fully capture psychological contract dynamics within service delivery processes. Third, the coupling coordination degree model predominantly emphasizes static inter-system interactions and provides limited explanatory power for nonlinear dynamic feedback mechanisms between supply and demand. Moreover, although the entropy method enhances objectivity in weight determination, it remains subject to algorithmic constraints inherent in quantitative weighting approaches. Finally, although the eight-year panel offers relatively long-term observation, certain health outcome indicators characterized by substantial lag effects may not be fully captured within the study period. Future research should incorporate spatial econometric techniques and dynamic modeling frameworks to facilitate deeper structural diagnostics.

Looking ahead, primary-level medical–prevention integration will continue to advance under the combined impetus of national strategy, technological empowerment, workforce development, and life-course health management. The China central and local governments are expected to further strengthen top-level policy design and fiscal investment, while refining incentive and compensation mechanisms for integration. From a technological perspective, digital health tools—including internet-based medical services and electronic health records—should be leveraged to eliminate information silos between clinical and public health systems, enable real-time data sharing, and enhance the precision of disease surveillance and intervention. Intelligent healthcare platforms and big data analytics will increasingly function as pivotal instruments for reinforcing integration at the primary level. In terms of human resources and organizational innovation, the relatively low proportion of general practitioners and persistent shortages of grassroots healthcare personnel remain critical bottlenecks. Accelerating the cultivation of comprehensive competencies and interprofessional collaboration capacity among primary healthcare workers will be essential. Institutional mechanisms such as regional medical consortia should be further developed to incentivize the downward flow of medical and preventive resources into community settings. Concurrently, with the deepening of population ageing, life-course health management will assume growing prominence. Routine chronic disease screening, health interventions, and immunization programs are expected to be more systematically integrated with regular clinical services, fostering a prevention-oriented primary healthcare service system characterized by seamless coordination between medical treatment and public health functions.

In summary, with sustained top-level policy support and continued practical innovation, primary-level medical–prevention integration in Hebei Province and across China is poised to evolve from formal coupling toward substantive service coordination. Through ongoing institutional reform and optimized resource allocation, a new health management paradigm featuring organic integration and continuous service coordination can be established, thereby delivering more precise and efficient healthcare services and consolidating the grassroots foundation of the Healthy China strategy.

## Supporting information

S1 Table Integrated Medical and Preventive Coordination Index of Township/Community Health Institutions in Hebei’s Prefecture-level Cities from 2017 to 2024（α = 0.4，β = 0.6）. (DOCX)

S2 Table Integrated Medical and Preventive Coordination Index of Township/Community Health Institutions in Hebei’s Prefecture-level Cities from 2017 to 2024（α = 0.6，β = 0.4）. (DOCX)
