# Supplementary material for: Evaluation of coupling and coordination, obstacle diagnosis, and optimization pathways of medical–preventive integration in primary healthcare institutions in Hebei Province from 2017 to 2024
Source: PLoS One. 2026 Jun 30;21(6):e0351931. doi: 10.1371/journal.pone.0351931 (PMC13318188; doi:10.1371/journal.pone.0351931)
Supplement: S1 Table — (DOCX) [file pone.0351931.s001.docx]

**Supporting information**

**S1 Table. Integrated Medical and Preventive Coordination Index of Township and Community Health Institutions in Hebei’s Prefecture-level Cities from 2017 to 2024 (α = 0.4, β = 0.6)**

| Region | 2017 | | | 2018 | | | 2019 | | | 2020 | | | 2021 | | | 2022 | | | 2023 | | | 2024 | |
| --- | --- | --- | --- | --- | --- | --- | --- | --- | --- | --- | --- | --- | --- | --- | --- | --- | --- | --- | --- | --- | --- | --- | --- |
|  | X S | | | X S | | | X S | | | X S | | | X S | | | X S | | | X S | | | X S | |
| Shijiazhuang | 0.531 | 0.161 | 0.552 | | 0.171 | 0.550 | | 0.189 | 0.557 | | 0.167 | 0.476 | | 0.146 | 0.477 | | 0.171 | 0.546 | | 0.242 | 0.584 | | 0.261 |
| Tangshan | 0.400 | 0.085 | 0.422 | | 0.088 | 0.422 | | 0.103 | 0.386 | | 0.097 | 0.419 | | 0.101 | 0.385 | | 0.103 | 0.434 | | 0.128 | 0.442 | | 0.141 |
| Qinhuangdao | 0.178 | 0.043 | 0.225 | | 0.048 | 0.211 | | 0.055 | 0.176 | | 0.074 | 0.175 | | 0.080 | 0.185 | | 0.043 | 0.242 | | 0.057 | 0.236 | | 0.080 |
| Handan | 0.620 | 0.059 | 0.604 | | 0.047 | 0.554 | | 0.062 | 0.540 | | 0.055 | 0.536 | | 0.054 | 0.501 | | 0.100 | 0.555 | | 0.091 | 0.587 | | 0.091 |
| Xingtai | 0.458 | 0.057 | 0.438 | | 0.052 | 0.402 | | 0.048 | 0.379 | | 0.054 | 0.401 | | 0.057 | 0.461 | | 0.062 | 0.558 | | 0.068 | 0.577 | | 0.082 |
| Baoding | 0.614 | 0.103 | 0.605 | | 0.112 | 0.557 | | 0.105 | 0.524 | | 0.090 | 0.432 | | 0.073 | 0.412 | | 0.084 | 0.470 | | 0.103 | 0.505 | | 0.129 |
| Zhangjiakou | 0.250 | 0.082 | 0.274 | | 0.090 | 0.248 | | 0.091 | 0.230 | | 0.058 | 0.230 | | 0.066 | 0.227 | | 0.061 | 0.250 | | 0.075 | 0.249 | | 0.072 |
| Chengde | 0.321 | 0.052 | 0.313 | | 0.096 | 0.289 | | 0.097 | 0.261 | | 0.092 | 0.286 | | 0.088 | 0.295 | | 0.077 | 0.314 | | 0.082 | 0.304 | | 0.083 |
| Cangzhou | 0.389 | 0.072 | 0.391 | | 0.069 | 0.383 | | 0.064 | 0.363 | | 0.054 | 0.376 | | 0.053 | 0.382 | | 0.037 | 0.390 | | 0.048 | 0.406 | | 0.051 |
| Langfang | 0.256 | 0.046 | 0.214 | | 0.044 | 0.208 | | 0.054 | 0.198 | | 0.033 | 0.200 | | 0.042 | 0.205 | | 0.061 | 0.227 | | 0.075 | 0.262 | | 0.084 |
| Hengshui | 0.227 | 0.020 | 0.240 | | 0.031 | 0.227 | | 0.031 | 0.208 | | 0.030 | 0.219 | | 0.030 | 0.222 | | 0.035 | 0.238 | | 0.037 | 0.260 | | 0.077 |

Note: X represents Township Health Centers; S represents Community Health Service Centers.
