# Supplementary material for: Evaluation of coupling and coordination, obstacle diagnosis, and optimization pathways of medical–preventive integration in primary healthcare institutions in Hebei Province from 2017 to 2024
Source: PLoS One. 2026 Jun 30;21(6):e0351931. doi: 10.1371/journal.pone.0351931 (PMC13318188; doi:10.1371/journal.pone.0351931)
Supplement: S2 Table — (DOCX) [file pone.0351931.s002.docx]

**Supporting information**

**S2 Table. Integrated Medical and Preventive Coordination Index of Township and Community Health Institutions in Hebei’s Prefecture-level Cities from 2017 to 2024 (α = 0.6, β = 0.4)**

| Region | 2017 | | | 2018 | | | 2019 | | | 2020 | | | 2021 | | | 2022 | | | 2023 | | | 2024 | |
| --- | --- | --- | --- | --- | --- | --- | --- | --- | --- | --- | --- | --- | --- | --- | --- | --- | --- | --- | --- | --- | --- | --- | --- |
|  | X S | | | X S | | | X S | | | X S | | | X S | | | X S | | | X S | | | X S | |
| Shijiazhuang | 0.553 | 0.150 | 0.569 | | 0.159 | 0.569 | | 0.178 | 0.580 | | 0.159 | 0.502 | | 0.141 | 0.497 | | 0.164 | 0.546 | | 0.225 | 0.581 | | 0.242 |
| Tangshan | 0.413 | 0.075 | 0.432 | | 0.079 | 0.433 | | 0.093 | 0.401 | | 0.087 | 0.436 | | 0.091 | 0.399 | | 0.094 | 0.433 | | 0.116 | 0.440 | | 0.128 |
| Qinhuangdao | 0.175 | 0.040 | 0.214 | | 0.044 | 0.202 | | 0.051 | 0.174 | | 0.066 | 0.174 | | 0.071 | 0.179 | | 0.042 | 0.227 | | 0.053 | 0.220 | | 0.072 |
| Handan | 0.633 | 0.052 | 0.615 | | 0.042 | 0.566 | | 0.060 | 0.553 | | 0.054 | 0.553 | | 0.055 | 0.522 | | 0.105 | 0.560 | | 0.087 | 0.583 | | 0.089 |
| Xingtai | 0.468 | 0.056 | 0.445 | | 0.053 | 0.415 | | 0.049 | 0.393 | | 0.055 | 0.413 | | 0.058 | 0.471 | | 0.062 | 0.534 | | 0.068 | 0.556 | | 0.079 |
| Baoding | 0.643 | 0.099 | 0.637 | | 0.105 | 0.592 | | 0.101 | 0.572 | | 0.091 | 0.466 | | 0.073 | 0.442 | | 0.083 | 0.488 | | 0.099 | 0.516 | | 0.120 |
| Zhangjiakou | 0.246 | 0.072 | 0.268 | | 0.079 | 0.244 | | 0.082 | 0.229 | | 0.054 | 0.230 | | 0.060 | 0.227 | | 0.055 | 0.244 | | 0.067 | 0.245 | | 0.067 |
| Chengde | 0.307 | 0.045 | 0.300 | | 0.080 | 0.280 | | 0.080 | 0.257 | | 0.076 | 0.280 | | 0.072 | 0.285 | | 0.064 | 0.296 | | 0.068 | 0.288 | | 0.070 |
| Cangzhou | 0.425 | 0.064 | 0.427 | | 0.062 | 0.420 | | 0.059 | 0.400 | | 0.051 | 0.413 | | 0.052 | 0.417 | | 0.038 | 0.419 | | 0.049 | 0.431 | | 0.053 |
| Langfang | 0.263 | 0.039 | 0.220 | | 0.038 | 0.219 | | 0.049 | 0.212 | | 0.033 | 0.214 | | 0.040 | 0.217 | | 0.055 | 0.235 | | 0.069 | 0.265 | | 0.077 |
| Hengshui | 0.237 | 0.018 | 0.247 | | 0.028 | 0.237 | | 0.031 | 0.221 | | 0.030 | 0.232 | | 0.029 | 0.234 | | 0.033 | 0.244 | | 0.035 | 0.263 | | 0.076 |

Note: X represents Township Health Centers; S represents Community Health Service Centers.
